# Epidemiology of injuries and illnesses in elite wheelchair basketball players over a whole season – a prospective cohort study

**DOI:** 10.1186/s13102-023-00692-6

**Published:** 2023-07-14

**Authors:** Moritz Weith, Astrid Junge, Tim Rolvien, Sascha Kluge, Karsten Hollander

**Affiliations:** 1 Department of Trauma and Orthopedic Surgery, Division of Orthopedics, University Medical Center Hamburg-Eppendorf, Martinistraße 52, Hamburg, 20246 Germany; 2 University of Bonn Medical Center, Venusberg-Campus 1, Bonn, 53127 Germany; 3 Institute of Interdisciplinary Exercise Science and Sports Medicine, MSH Medical School Hamburg, Am Kaiserkai 1, Hamburg, 20457 Germany; 4 Center for Health in Performing Arts, MSH Medical School Hamburg, Am Kaiserkai 1, Hamburg, 20457 Germany; 5 Zentrum für Rehabilitationsmedizin, BG Klinikum Hamburg, Bergedorfer Straße 10, Hamburg, 21033 Germany

**Keywords:** Paralympic sports, Wheelchair basketball, Injury surveillance, Epidemiology, Health monitoring, Injury prevention

## Abstract

**Background:**

Wheelchair basketball is an adaptation of pedestrian basketball and one of the most popular Paralympic sports worldwide. The epidemiology of health problems in wheelchair basketball has been prospectively studied only during the Paralympic Games, the 2018 World Championships, the 2021 South America Wheelchair Basketball Championship, and one season of two American intercollegiate wheelchair basketball teams. The objective of the study was to prospectively monitor and analyze the prevalence, incidence, burden, and characteristics of injuries and illnesses in a wheelchair basketball league during an entire season for the first time.

**Methods:**

All players of the highest German wheelchair basketball league (Bundesliga) were invited to participate in the study. Included players completed the Oslo Sports Trauma Research Center Questionnaire once a week during the entire season 2020/21 to report health problems. Exposure was captured by self-reported training time and officially-recorded competition time.

**Results:**

Sixty of 117 players (51%, 47 male, 13 female) of the national league participated with an average response of 93%. Seventy health problems (5.5/1000 exposure hours [95% CI: 4.9–6.1]) were reported, including 54 injuries and 16 illnesses. Prevalence of health problems was 60% (95% CI: 48–72). Most injuries affected the shoulder (32% of all injuries), cervical spine/neck (17%), and hand (13%). More overuse injuries (2.9/1000 exposure hours [95% CI: 2.5–3.3]) than acute injuries (1.3/1000 exposure hours [95% CI: 1.0-1.6]) occurred. Of all health problems, 53% were associated with time-loss. The incidences of all health problems, illnesses, injuries, and overuse injuries were higher in women than in men.

**Conclusions:**

Characteristics and frequency of injuries and illnesses during wheelchair basketball season differed from those during major wheelchair basketball tournaments. The high proportion of overuse injuries and the higher injury rates in women should be regarded in the development of individualized prevention measures. Since results from previous studies during major tournaments are only partially comparable to wheelchair basketball league play, further studies should follow.

## Background

Wheelchair basketball (WB) is an adaption of able-bodied basketball and one of the most popular adaptive sports worldwide. It was first played by World War II veterans in the United States of America in 1945 and spread constantly in the years after. Since 1960 it is part of the Paralympic Games [1] and since the early 1970’s there are officially organized WB competitions in Germany. Nowadays, there is a multi-level league system with a national league (Bundesliga) at its top.

While injuries and illnesses have been intensively researched in Olympic sports, [2–5] there is a lack of knowledge in Paralympic sports. The first published study about health problems in Paralympic sports showed that WB players were the second most injured athletes compared to other wheelchair sports [6]. During the 2012 and 2016 Summer Paralympics, injuries were systematically recorded [7, 8]. Incidence rates of 12 (95% confidence interval [95% CI]: 8.3–16.8) [7] and 12.8 injuries per 1000 athlete-days (95% CI: 9.5–17.4) [8] were reported in WB. In 2018, Hollander et al. [9] monitored sports injuries during the World Championships for the first time. The injury rate was 68.9 injuries per 1000 athlete-days (95% CI: 55.4–82.4), with injuries of the neck/cervical spine, the thoracic spine/upper back, and the shoulder being the most common [9].

The recording of health problems in the context of major international tournaments represents only a small part of an athlete’s life. In a retrospective survey, Kluge et al. [10] found that 46% of the participants in the 2018 World Championships had at least sometimes health problems in the year before the tournament, however, no further details about these health problems were recorded. Kasitinon et al. [11] monitored health problems of intercollegiate WB players during one competitive season. They concluded that the injury risk was significantly higher for WB players than for non-disabled intercollege basketball players in the National Collegiate Athletic Association (NCAA). This study was the first to include WB players who were not part of a national team, but only players from two teams from a single site were considered [11].

In non-disabled sports, such as basketball, prospective recording of health problems in national leagues is well established [12–14] and a prerequisite to better understanding health problems and their characteristics.

The aim of the present study was to prospectively monitor and analyze the prevalence, incidence, burden, and characteristics of health problems (injuries and illnesses) in the WB Bundesliga throughout an entire season. A secondary aim of the study was to compare the results of male and female players.

## Methods

### Study design, population, and setting

This prospective cohort study recorded health problems in the highest German WB league (Bundesliga) weekly during the season 2020/21 using an established online reporting tool (AthleteMonitoring, FitStats Technologies, Moncton, Canada). The season started on the 31st of October 2020 and finished on the 15th of May 2021. Regularly, ten teams compete against each other over a period of 29 weeks, and all teams play a home round and an away round. The teams are mixed, meaning that women and men play in the same team. The four best teams qualify for the playoffs. Depending on their qualification for the semifinal or the final of the playoffs, they play two to six games in the playoffs. Players from three teams additionally participated in international competitions. The exposure and health problems that occurred during international competitions were treated the same as the data collected during the league. Due to the COVID-19 pandemic, only seven teams started participating in the league on the 31st of October 2020 and played the full season matches, while three teams did not participate in the league until January 2021 and played only the games of the return round.

The study was introduced to the teams for the first time at the league conference in June 2020. At this conference one representative of each team was present. Between September 2020 and the start of the season, a member of the research team presented the study to each team that was willing to participate in detail face-to-face or a video conference. All players who competed in the national league were invited to participate in the study, irrespective of sex and age. Thus, also minor-aged players were included in the study. Informed written consent has been obtained from all participants or legal guardians of minor-aged participants. All participants received detailed information on the study including how to use the online reporting tool, fill in the questionnaire, and contact data of the research group.

### Data collection

During the complete season injuries and illnesses were tracked weekly using the Oslo Sports Trauma Research Center Questionnaire on Health Problems (OSTRC-H2) in its updated and validated English [15] and German version [16]. The questionnaire was sent to the participants by using the online reporting tool AthleteMonitoring (FitStats Technologies, Moncton, Canada). The OSTRC-H2 consists of four questions on whether and to what extent participation in training/competition, training volume and performance was limited, and whether and to what extent symptoms occurred in the past seven days due to a health problem. Furthermore, the participants were asked to report the type of health problem (acute injury, overuse injury, illness) and the body region of injuries. Moreover, they could record multiple health problems and provide additional free-text comments.

Through personal contact, the research group tried to motivate weekly compliance with questionnaire submission and was available in case of questions or technical issues. The period of data collection covered a total of 29 weeks. Those athletes who started their season later (due to the COVID-19 pandemic) or finished it earlier (because they did not qualify for the playoffs, were eliminated from the playoffs, or ended their participation in the study earlier) were only included in the study as long as they were competing in the league/were willing to participate in the study. As recommended by Clarsen et al. [17], responses to the first OSTRC-H2 questionnaire sent out the week before the season started were excluded from the final data set. To track training exposure, the participants were asked every week how long they had practiced in the last seven days. Game exposure was collected from International Basketball Federation (FIBA) LiveStats [18].

In addition, a one-time survey before the start of the season was used to record the sex, age, and disability classification of the participants. The disability classification is based on a point system ranging from 1.0 to 4.5 points according to the player’s disability. A low score is associated with higher limitations in terms of body stability and balance [19].

### Definition of injury and illness

The OSTRC-H2 defines a health problem as follows: “A health problem is any condition that you consider to be a reduction in your normal state of full health, irrespective of its consequences on your sports participation or performance, or whether you have sought medical attention. This may include, but is not limited to, injury, illness, pain or mental health conditions.” [15] Health problems is the generic term for all kind of injuries and illnesses. Acute injuries were defined as injuries resulting from a single, identifiable event. Injuries with gradual-onset were defined as overuse injuries [20]. Substantial health problems are defined as those leading to moderate or severe reductions in training volume, or moderate or severe reductions in sports performance, or complete inability to participate in sport (problems where athletes selected option 3, 4, or 5 in either questions 2 or 3) [21].

### Data analysis

The rate of health problems during the season was calculated as incidence rate, season prevalence, weekly prevalence, and average weekly prevalence. Total exposure was the sum of training time and game time in hours. As training time and game time were tracked individually the exact exposure time was calculated. The incidence rate of health problems and of injuries was calculated using the formula: (number of health problems / exposure time) * 1000 and was expressed as the number of health problems per 1000 hours of exposure. The incidence rate of illnesses was calculated per athlete-days. Incidence rates were calculated based on all health problems that newly occurred during the season [22]. To calculate 95% CIs the following formula was used: incidence rate ± 1.96 x √(incidence rate x (1 – incidence rate) / exposure time). Prevalence was calculated using the formula: (number of affected athletes during a time period / number of exposed athletes) x 100 and was expressed as percentage of affected athletes. It was calculated based on all injured or ill players during a specified period of time (season or week), including health problems that were incurred before the beginning of the season [22]. To calculate 95% CIs the following formula was used: prevalence ± 1.96 x √(prevalence x (1 – prevalence) / number of exposed athletes).

The answers to each of the four questions of the OSTRC-H2 questionnaire were assigned values between 0 and 25, with 0 representing no problems and 25 the maximum level. The sum of these values results in an ordinally scaled severity score between 0 and 100 [17]. The cumulative severity score was defined as the sum of the weekly severity scores reported on the same health problem [22]. The burden as a measure of the impact of health problems was defined as the sum of the cumulative severity scores of all health problems in a subgroup (for example, all overuse injuries) [22, 23]. The relative burden as a proportion of the total burden was calculated as the sum of the cumulative severity scores for all health problem types divided by the cumulative severity score for all health problems [23].

Differences between male and female players were analyzed. Potential group differences were tested using Pearson chi-square tests. For the data analysis, Microsoft Excel version 16.49 and IBM SPSS Statistics version 28.0.1.0 were used. All information was treated strictly confidential, and all data were stored on laptops that were secured by passwords. Ethical approval was granted by the Ethics Committee of the University of Hamburg (protocol number AZ 2020_291). The study is reported according to the Strengthening the Reporting of Observational Studies in Epidemiology (STROBE) [24] guidelines for cohort studies.

## Results

### Participants and exposure data

Ten teams with a total of 117 players (94 male, 80%) competed in the German national league’s season 2020/21. More than half of the players (n = 60, 51%) from eight different teams participated in the study. The participants were mainly male (n = 47; 78%) and had a mean age of 27.7 (SD ± 6.3) years. One participant terminated his participation in the study earlier but allowed us to use the data collected to that point.

All disability classifications were represented. The study population was similar to the entire league regarding sex and disability classification (Table 1). Data on age were not available for players who did not participate in the study.

**Table 1:** Characteristics of all athletes in the league and all study participants

Number of	Complete league n (%)	Study population n (%)
**Teams**	10	8
**Athletes**		
Total	117	60
Men	94 (80)	47 (78)
Women	23 (20)	13 (22)
**Disability classification**		
1.0	21 (18)	14 (23)
1.5	6 (5)	2 (3)
2.0	14 (12)	5 (8)
2.5	4 (3)	2 (3)
3.0	19 (16)	13 (22)
3.5	9 (8)	1 (2)
4.0	9 (8)	4 (7)
4.5	35 (30)	19 (32)

The average response rate to the weekly questionnaire was 93% (range: 86-98%). A total of 254 match-hours and 12,432 practice-hours were reported throughout the season. The average total exposure per player was 211.4 h, with an average weekly exposure of 11.6 h per player. The average weekly exposure of female participants (12.3 h (95% CI: 11.3–13.4)) was slightly higher than that of males (11.4 h (95% CI: 11.0-11.8)).

### Number, prevalence, and incidence of health problems

Over the season, 70 health problems were reported by 26 men and 10 women. Twenty-four participants (40% [95% CI: 28–52]) reported no health problem during the season, 14 (23% [95% CI: 13–34]) one health problem, 22 (37% [95% CI: 25–49]) two or more health problems, and 7 (12% [95% CI: 4–20]) three or more. On average, women reported 2.5 health problems (95% CI: 0.9–4.2), men 1.0 health problem (95% CI: 0.7–1.3).

Most health problems (77%) were injuries (32 in men, 22 in women) and 16 illnesses (men 9, women 7). About two thirds of injuries were classified as overuse (men 19, women 18), and 17 (32% of all injuries) as acute (men 13, women 4).

**Fig. 1:**
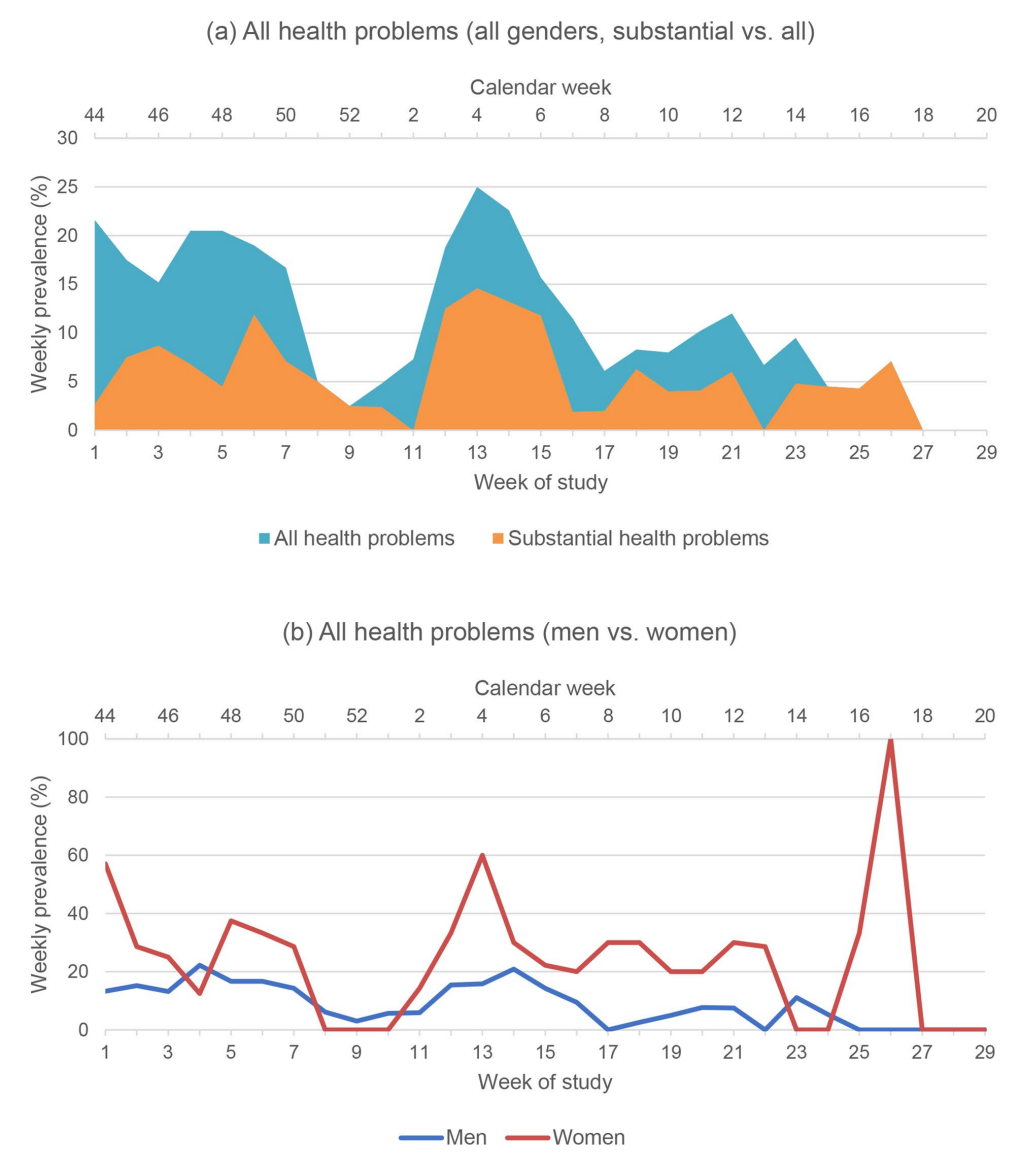
Weekly prevalence of all health problems/substantial health problems (**a**) and in men/women (**b**)

All prevalence and incidence rates are presented in Table 2. The season prevalence of health problems was 60% (95% CI: 48–72). For injuries it was 47% (95% CI: 34–59) and for illnesses 22% (95% CI: 11–32). The average weekly prevalence of health problems was 11% (95% CI: 1–21) and of substantial health problems 5% (95% CI: 0–13). Figure 1 shows the development of the weekly prevalence over the course of the season. The prevalence of illnesses was significantly higher in women than in men (Chi-square 10.1, p = 0.001). No sex-specific differences were observed for the average weekly prevalence, the prevalence of all health problems, and of injuries.

The overall incidence of health problems was 5.5 per 1000 exposure-hours (95% CI: 4.9–6.1) (Table 2). The incidence of injuries was 4.3 per 1000 exposure-hours (95% CI: 3.7–4.8). The incidence of overuse injuries (2.9 per 1000 exposure-hours [95% CI: 2.5–3.3]) was about twice as high as that of acute injuries (1.3 per 1000 exposure-hours [95% CI: 1.0-1.6]). The incidence of illnesses was 2.1 per 1000 athlete-days (95% CI: 1.7–2.4). As shown in Table 2, the incidences of all health problems, illnesses, all injuries, and overuse injuries were significantly higher in women than in men. There was no sex-specific difference in the incidence of acute injuries.

**Table 2:** Incidence, prevalence and average weekly prevalence of health problems in male, female and all players

Incidence	Total	95% CI	Men	95% CI	Women	95% CI
**All health problems**						
per 1000 exposure-hours	5.5	4.9–6.1	4	3.4–4.6	12.1	10.2–13.9
**Illnesses**						
per 1000 athlete-days	2.1	1.7–2.4	1.4	1.1–1.8	5.1	3.9–6.4
**Injuries**						
per 1000 exposure-hours	4.3	3.7–4.8	3.1	2.6–3.6	9.2	7.5–10.8
**Overuse injuries**						
per 1000 exposure-hours	2.9	2.5–3.3	1.8	1.5–2.2	7.5	6.0–9.0
**Acute injuries**						
per 1000 exposure-hours	1.3	1.0-1.6	1.3	0.9–1.6	1.7	1.0-2.4
**Prevalence**	**Total (%)**	**95% CI**	**Men (%)**	**95% CI**	**Women (%)**	**95% CI**
**All health problems**	60	48–72	55	41–70	77	54–100
**Illnesses**	22	11–32	13	3–22	54	27–81
**Injuries**	47	34–59	45	31–59	54	27–81
Overuse injuries	37	25–49	36	22–50	39	12–65
Acute injuries	18	9–28	17	6–28	23	0–46
**Multiple health problems** (more than 1 health problem reported)	37	25–49	32	19–45	54	27–81
**Average weekly prevalence**	**Total (%)**	**CI 95%**	**Men (%)**	**CI 95%**	**Women (%)**	**CI 95%**
**All health problems**	11	1–21	9	0–18	24	0–56
**Illnesses**	2	0–6	1	0–5	5	0–21
**Injuries**	9	0–18	7	0–16	19	0–49
Overuse injuries	6	0–14	5	0–13	10	0–33
Acute injuries	3	0–8	2	0–7	9	0–30
**Substantial health problems**	5	0–13	5	0–12	11	0–35

### Location of injury

The injured body parts for all, acute and overuse injuries, time-loss and non-time-loss injuries, and injuries in male and female players are presented in Table 3. The shoulder (32%), the cervical spine/neck (17%), and the hand (13%) were the most common injury locations. Acute injuries occurred most frequently at the shoulder, hand, and pelvis (18% each) and chest/ribs/upper back (12%). Two-thirds of all injuries, 78% of overuse, and 40% of acute injuries at the upper extremity affected the players’ dominant side.

**Table 3:** Location of injuries

	All injuries		Overuse injuries		Acute injuries		Injuries in men		Injuries in women		Time-loss injuries		Non time-loss injuries	
	n	%	n	%	n	%	n	%	n	%	n	%	n	%
**Head**	1	2	0	0	1	6	0	0	1	5	1	4	0	0
**Cervical spine/neck**	9	17	8	22	1	6	5	16	4	18	5	22	4	13
**Chest/ribs/upper back**	4	7	2	5	2	12	2	6	2	9	1	4	3	10
**Lower back**	1	2	1	3	0	0	1	3	0	0	1	4	0	0
**Abdomen**	0	0	0	0	0	0	0	0	0	0	0	0	0	0
**Shoulder**	17	32	14	38	3	18	10	31	7	32	6	26	11	36
**Upper arm**	1	2	0	0	1	6	1	3	0	0	1	4	0	0
**Elbow**	1	2	0	0	1	6	0	0	1	5	1	4	0	0
**Forearm**	4	7	3	8	1	6	2	6	2	9	1	4	3	10
**Wrist**	3	6	2	5	1	6	2	6	1	5	1	4	2	7
**Hand**	7	13	4	11	3	18	4	13	3	14	3	13	4	13
**Pelvis**	4	7	1	3	3	18	3	9	1	5	1	4	3	10
**Hip**	0	0	0	0	0	0	0	0	0	0	0	0	0	0
**Thigh**	1	2	1	3	0	0	1	3	0	0	0	0	1	3
**Knee**	0	0	0	0	0	0	0	0	0	0	0	0	0	0
**Lower leg**	0	0	0	0	0	0	0	0	0	0	0	0	0	0
**Ankle**	0	0	0	0	0	0	0	0	0	0	0	0	0	0
**Foot**	1	2	1	3	0	0	1	3	0	0	1	4	0	0

### Severity

More than half (53%) of all reported health problems resulted in time-loss (88% of all illnesses, 43% of all injuries). The average time-loss of all time-loss health problems was 4.7 days, ranging between 1 day and 28 days. Acute injuries caused 38% of all time-loss days, illnesses 36%, and overuse injuries 26%.

All details of average weekly severity scores are shown in Table 4. The largest sex difference was found for overuse injuries (men 47.5, women 29.9), and the smallest for acute injuries (men 64.7, women 64.5).

**Table 4:** Duration, average weekly severity score, cumulative severity score, and average time-loss of health problems

	All players	Interquartile Range	Men	Interquartile Range	Women	Interquartile Range
**All health problems**						
Duration (weeks)	1.5	1.0–2.0	1.7	1.0–2.0	1.3	1.0–1.0
Average weekly severity score	51.4	24.0–68.0	57.1	33.0–77.0	41.0	24.0–67.0
Cumulative severity score	77.1	33.0-100.0	94.7	41.0-115.0	52.3	24.0–68.0
Average time-loss (days)	4.7	1.0–5.0	5.2	1.0–5.0	3.7	2.0-4.3
**Illnesses**						
Duration (weeks)	1.3	1.0-1.3	1.4	1.0–2.0	1.1	1.0–1.0
Average weekly severity score	65.0	41.0–92.0	71.1	41.0-100.0	55.3	41.0-69.3
Cumulative severity score	85.4	41.0-106.8	102.7	41.0-127.0	63.1	41.0-75.5
Average time-loss (days)	4.4	1.3-4.0	5.9	1.0-8.8	2.5	2.0-3.5
**Overuse injuries**						
Duration (weeks)	1.6	1.0–2.0	1.8	1.0–3.0	1.3	1.0–1.0
Average weekly severity score	40.5	24.0–57.0	47.5	32.0-67.5	29.9	20.0-32.5
Cumulative severity score	63.5	24.0–68.0	87.5	40.5–128.0	38.2	24.0-32.8
Average time-loss (days)	3.5	1.0–5.0	3.2	1.0–5.0	5.5	5.3–5.8
**Acute injuries**						
Duration (weeks)	1.5	1.0–2.0	1.5	1.0–2.0	1.5	1.0–2.0
Average weekly severity score	64.7	50.0–96.0	64.7	45.8–100.0	64.5	61.3–68.0
Cumulative severity score	98.9	58.0-100.0	99.5	51.0-100.0	96.8	68.0-108.8
Average time-loss (days)	6.6	2.3-8.0	8.0	2.0-8.8	4.5	3.0-4.5

### Burden of health problems

Considering the cumulative severity scores as the measure of the burden of health problems, overuse injuries caused 44% of the total burden, acute injuries 31%, and illnesses 25%. Similar patterns emerged in health problems of men (overuse injuries 43%, acute injuries 33%, illnesses 24%) and women (overuse injuries 45%, illnesses 29%, acute injuries 26%). The relative burden of time-loss health problems was 77% of the total burden of all health problems.

**Fig. 2:**
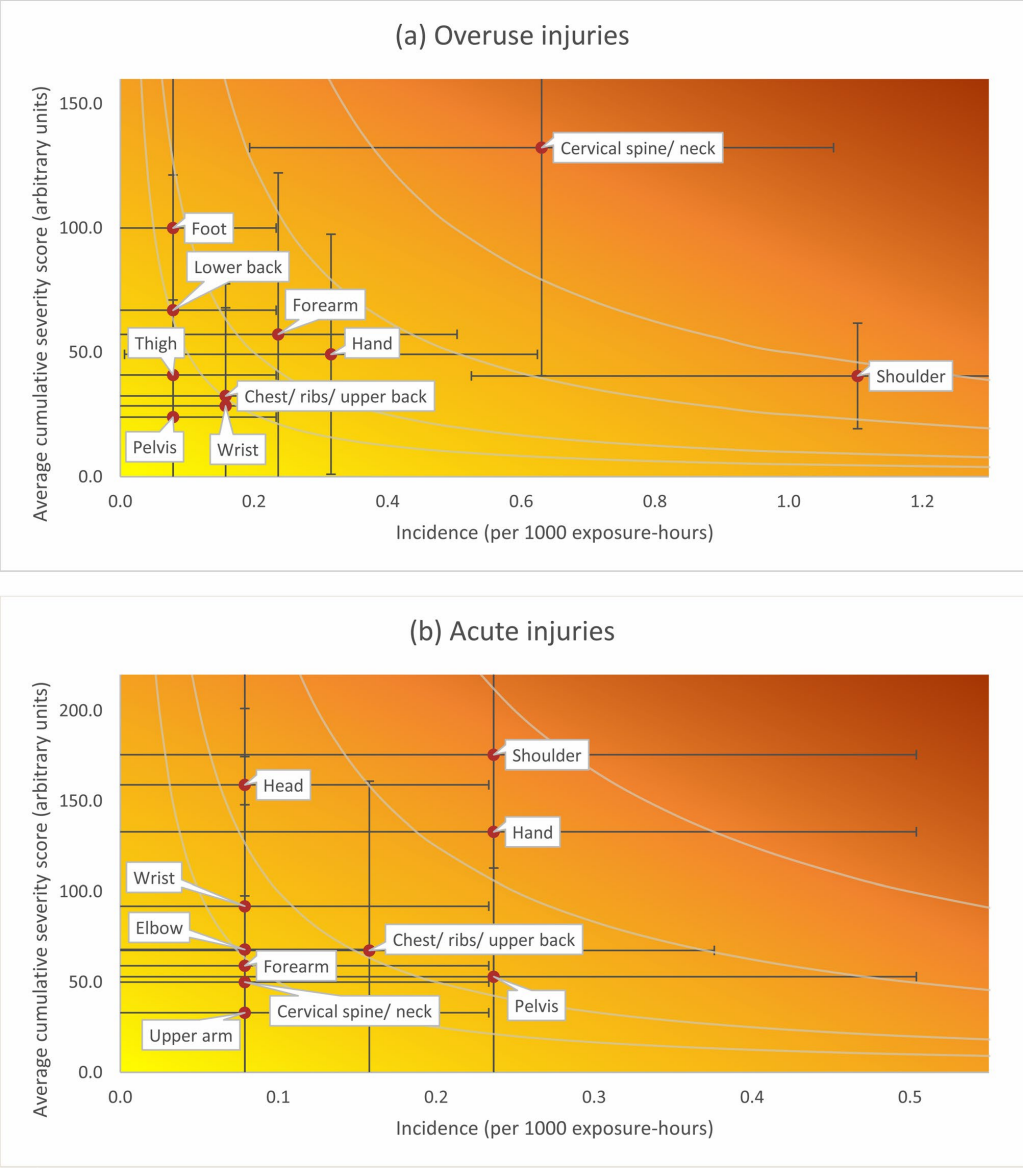
Relationship between severity and incidence for overuse (**a**) and acute injuries (**b**). The darker the color the greater the burden. The curved grey isobars represent points with equal burden. Vertical and hori-zontal error bars represent 95% confidence intervals. Regions with no injuries reported are not included in the figure

Figure 2 shows the relationship between severity and incidence for the body regions that were mostly affected by acute and overuse injuries. In acute injuries, the shoulder and hand represented the most burdensome locations, while for overuse injuries it was the shoulder and cervical spine/neck.

## Discussion

This study is the first to prospectively monitor health problems in WB players from multiple teams of a national league throughout one complete season. The season prevalence of health problems was 60%. The incidences of all health problems, illnesses, injuries, and overuse injuries were higher in female than in male players. Overall, overuse injuries were more common than acute injuries. Almost two thirds of all injuries occurred to the shoulder, cervical spine/neck, and hand. More than half of all health problems were associated with time-loss.

### Prevalence of health problems

On average 11% of all participants reported a health problem in each week, which is lower than in the German [25] (28%) and Norwegian Paralympic team [26] (37%). In the study on the Norwegian Paralympic team, numerous athletes from sports that are known to be injury-intensive participated (especially ice sledge hockey) [27]. In the investigation of the German Paralympic team, apart from WB players, mainly athletes from sports with low injury rates [8] participated. Therefore, the difference between the three methodologically similar studies cannot be clearly explained at present and further long-term investigations in Paralympic sports are needed.

60% of the participants reported at least one health problem during the season. During a retrospective survey of WB players participating in a World Championship, 46% reported having at least sometimes physical complaints in the past 12 months [10]. The retrospective, rather than prospective, survey, may be a reason for the divergence. But both studies show that health problems are part of the athlete’s life for a relevant proportion of WB players.

Almost half of the participants (47%) reported an injury during the season. From pedestrian basketball, a higher injury prevalence of 68% was reported [13]. In contrast to American intercollegiate WB, [11] based on our results, WB appears to be less injurious than pedestrian basketball [13]. The reason for the difference could be that only national league WB players participated in our study, while also players from lower leagues participated in the study in pedestrian basketball. These had a higher risk of injury than national league players. An injury incidence for national league players only was not reported. Future studies and studies in lower WB leagues should be awaited to allow a proper comparison with pedestrian basketball.

### Comparison of male and female athletes

In our cohort, the incidences of all health problems, illnesses, injuries, and overuse injuries were higher in women than in men except for the incidences of acute injuries. In contrast, Zech et al. [28] showed that in other team sports, men reported more overall injuries than women. Previous studies on WB players have so far not shown a clear sex difference. For example, in the 2018 World Championships, although women reported more overall health problems, there was no difference in overuse injuries [9]. There was also no sex-related difference in American intercollegiate WB players regarding the overall incidence of health problems, but women reported more gradual-onset injuries, and men reported more sudden-onset injuries [11]. However, in both studies, men and women practiced separately. In the German national league, men and women practice and compete together in one team, possibly making it less likely to accommodate the different physical conditions of both sexes and resulting in more health problems in women. Furthermore, the Relative Energy Deficiency in Sport may also influence the incidence of injuries [29]. The term Relative Energy Deficiency in Sport is used for physiological and performance consequences such as low bone mineral density and hormonal imbalances [30]. It is related to a higher incidence of injuries [31]. However, there have been few studies on the topic in Paralympic sports and it seems possible that athletes are affected by Relative Energy Deficiency in Sport regardless of sex [30–32].

### Characteristics of injuries

In accordance with Kasitinon et al., [11] we found that a higher number of injuries than illnesses were reported. Of all injuries in our study, 69% were overuse injuries. Among American intercollegiate WB players, [11] 31% were sudden-onset with trauma, 29% sudden-onset without trauma, and 40% gradual-onset injuries. The sum of sudden-onset without trauma and gradual-onset injuries was 69%, similar to the proportion of overuse injuries in our study. In contrast, 52% overuse injuries were reported in the 2018 World Championships, [9] and 53.8% in the 2021 South America Wheelchair Basketball Championships [33]. The proportion of overuse injuries appears to be greater in WB league play than in major tournaments. Possibly, more acute injuries occur in major tournaments due to greater intensity of play and density of games. Nevertheless, we still assume that both, acute and overuse injuries play a relevant role in WB league play. Especially because overuse injuries had the largest burden with 44% of the total cumulative severity score, raising awareness of coaches and implementing methods to control training load might be useful to prevent injuries [17, 34].

Most injuries occurred at the shoulder, cervical spine/neck, and hand, which is consistent with the results of previous studies of wheelchair athletes [9, 11, 29, 33, 35]. Overuse injuries were most common in the shoulder. Shoulder pain is common in wheelchair users, due to the daily need for wheelchair propulsion, even without participating in sports [36]. It can be therefore speculated that the reported shoulder overuse injuries were not only caused by the sport per se. García Gómez et al. [37] demonstrated that shoulder pain negatively affects throwing, especially in female WB players. Thus, the prevention of shoulder injuries not only serves to prevent injury but also to safeguard or even enhance performance. However, little is known about effective prevention measures to date [35]. Yuine et al. [38] showed in a first study that instability of the distal radioulnar joint leads to decreased hand function, such as upper arm/forearm strength. Therefore, future prevention measure development should not only focus on the shoulder but also other upper extremity joints.

### Time-loss rate

More than half of all health problems (53%) were associated with time-loss (88% of all illnesses, 43% of all injuries). In the context of major international tournaments, significantly fewer injuries were associated with time-loss, 8% during the 2018 World Championships [9] and 21.5% during the 2021 South America Wheelchair Basketball Championships,[33] respectively. Possibly, the reporting of many small, non-time-loss injuries such as muscle spasms and skin lacerations, especially in the 2018 World Championships [9] could explain the differences. Furthermore, an injury of the same severity in league play is presumably more likely to result in a time-loss due to the lower pressure to perform compared to major international tournaments. In American intercollegiate WB, 33% of all injuries, but only 57% of all illnesses, were associated with a time-loss [11]. Especially the difference in the time-loss rate of illnesses is striking here. The survey in American intercollegiate WB was conducted before the COVID-19 pandemic, whereas the present study was under the influence of the COVID-19 pandemic. Thus, it may be possible that training with disease symptoms is less frequent, due to higher awareness of the importance of infection risks and protecting teammates.

### Strengths and limitations

Strengths of our study are that 51% of all players in the league participated in the study and an average weekly response rate of 93% was achieved. In addition, the period of data collection was the longest ever in a study of WB, and the cohort was the largest in a prospective study of WB league play. Also, because the cohort was comparable to the complete league regarding sex and classification distribution, the results may be generalizable to the entire league. Since the exposure time was recorded individually and was not estimated, the incidences could be calculated more accurately [9, 11]. The questionnaire used was completed by the athletes without the assistance of medical personnel. On the one hand, this is a strength, as players were able to participate in the study without regular contact with medical staff.

On the other hand, the lack of medical examinations is the most important limitation of the study. As few details and no diagnoses of reported health problems were recorded, the definition of injuries and the distinction between acute injuries and overuse injuries might be inconsistent. Data should be compared to medical diagnoses in future studies, but national-level wheelchair basketball teams may not always be equipped with a medical team within their entourage. Furthermore, selection bias could be present because only some of the players in the Bundesliga are professional athletes. These might have had more capacity to participate in the study than those who have a working career on the side. Also due to the COVID-19 pandemic, fewer games than usual took place and only the league under study was able to play. As a result, the cohort was smaller than originally planned.

As known from previous studies, training conditions in WB are heterogeneous in different countries, [10] which is why the present results can be transferred to other countries and leagues only to a limited extent.

### Perspective

Due to COVID-19 pandemic restrictions, only the first Bundesliga took place in 2020/21. Therefore, methodologically similar studies should be conducted in other (lower) German and international leagues in the future. This would allow longitudinal data and data from larger cohorts to be collected, and the influence of potential predictors, such as sex, classification, age, and performance class, could be further investigated. Additionally, future studies should collect more details on reported health problems, such as sports activity, injury mechanism, etiology of illnesses, and diagnosis, to allow a more detailed analysis of the health problems that occurred and should continue to investigate whether there are differences in the characteristics and frequency of reported health problems over the course of the season.

## Conclusion

By recording health problems in league play over several months, this study examined a large part of the everyday sporting life of a WB player more comprehensively than previous prospective studies in WB. Due to the high proportion and large burden of overuse injuries, these could be a good foundation for future prevention measures. It should be taken into account that women reported more health problems than men, so developing different prevention measures for men and women may be appropriate. Nevertheless, the results of the study indicate that findings from previous studies in WB major tournaments and Paralympic sports are only partially transferable to WB league play. Therefore, the results suggest that further, methodically similar, research is needed in WB league play.

## Data Availability

Due to the confidentiality of the data collected, they are only available from the corresponding author on reasonable request.

## Abbreviations

WB: Wheelchair Basketball
95% CI: 95% confidence interval
OSTRC-H2: Oslo Sports Trauma Research Center Questionnaire on Health Problems
